# No conservation silver lining to Ebola

**DOI:** 10.1111/cobi.12454

**Published:** 2015-01-07

**Authors:** Simon Pooley, John E. Fa, Robert Nasi

**Affiliations:** ^1^ Imperial College Conservation Science, Department of Life SciencesImperial College LondonSilwood Park CampusBuckhurst RoadAscotSL5 7PYUnited Kingdom; ^2^ CIFOR and CGIAR ForestsTrees and Agroforestry programCIFOR HeadquartersJalan CIFORSitu GedeBogor16115Indonesia

In August 2014, the United Nations health authority declared the Ebola epidemic centered on Sierra Leone, Liberia, and Guinea an “international public health emergency” (WHO 2014). By October, public commentaries were omnipresent in print and online, including several statements in the mass media by wildlife conservationists. Their comments raise a number of uncomfortable issues about the consumption and trade of bushmeat in the region and in Africa more broadly that merit unpacking and rebuttal. The Ebola epidemic should not, in our view, be used as a Trojan horse to achieve wildlife conservation ends. This is both because some of the proposed conservation measures are of questionable efficacy, and may even backfire, and because doing so raises unfortunate associations with the long history of an outdated discourse of conservation in Africa that favored wildlife over people.

The most prominent conservation‐oriented response was the argument that clamping down on the consumption of and trade in wild animals (especially bats and primates) by Africans may be the key to preventing such epidemics (e.g., Williams 2014; Osofsky 2014; Young 2014). Jeffrey Stern implied in *Vanity Fair* that preventing deforestation would help retain “the buffer separating humans from animals and from the pathogens that animals harbour.” He argued that deforestation had driven bats in particular to rely on plantations rather than (disappearing) natural food sources for sustenance (Stern 2014; Young 2014).

Setting aside important arguments about values, not to mention the magnitude of the “protein gap” in many tropical regions (Fa et al. 2003), the practicality of stopping people from eating bushmeat deserves comment. We are concerned that in a time of “paranoia and uncertainty” in which we are seeing “behaviours reminiscent of those during the Black Death” (Williams’ interpretation of current circumstances in the region), Williams’ suggestion that fear be mobilized to prevent the eating of certain animals could backfire. It could lead to attempts to eradicate the vectors of the disease, not dissimilar to the support for the practice of culling of badgers in the United Kingdom from farmers who believe this will reduce tuberculosis in cattle (Raymond 2014). Thus, bats, chimpanzees, and other species that are primary sources of Ebola may become equally demonized; this would have the opposite effect to that Williams and Osofsky desire (as happened to wild urban primates in Brazil [Young 2014]). Equally, the habitats of fruit bats (remaining stands of natural forest) in the vicinities of human settlements could be targeted for destruction, as so many square kilometers of African bush were once cleared to prevent the spread of sleeping sickness.

This is not to argue that the consumption of bushmeat is not having a serious impact on the abundance of certain wildlife species in tropical regions (Milner‐Gulland et al. 2003). Basing their estimates on data from the Congo Basin, Fa et al. (2003) estimate that 4.9 million tons of wild mammal meat feeds millions of people living in Afrotropical forests annually. However, the consumption of some faster breeding species (such as large rodents or small duikers) that represent up to 70% of the bushmeat trade in West and Central Africa for subsistence purposes is not necessarily endangering these faster breeding species (Fa 2007; Fa & Brown 2009; Nasi et al. 2011). To maintain clarity over what human behavior threatens the survival of populations of wild animals and what does not, it is necessary to distinguish between taxa that can be hunted sustainably and taxa that are likely to be at greater risk from hunting. Doing so will also help avoid foisting particular culturally specific moral imperatives (not eating wild animals) on others from different cultural backgrounds and economic circumstances, not to mention valuing wild animals in Africa (should not be eaten) in a different way to valuing wild animals in the developed world, notably the United States and Europe (where they are widely eaten).

Eight years ago, a group of international wildlife conservation and development experts met in Jersey, United Kingdom, to form a consensus on the bushmeat crisis in Central and West Africa. They concluded that “[t]he ecological, nutritional, economic, and intrinsic values of wildlife hunted for food are all at risk of being lost because present policies and practices cannot reconcile these different values of bushmeat or manage the resource sustainably.” In some regions, the surviving wildlife species are mostly small, fast‐ reproducing species, and some require control measures because they are crop pests. In others, large‐bodied, slow‐reproducing species are being hunted to extinction. It is vital, the assembled experts agreed, “to understand when and where the bushmeat trade is primarily a livelihoods issue, a biodiversity conservation crisis, or both.” For the rural poor, all wildlife provides a safety net against short‐term livelihood crises, and subsistence use of the small, fast‐breeding species can be an important protein supplement to the human diet that has minimal conservation impacts (Bennett et al. 2006: 884, 885, 886).

We agree with Williams (2014) that one answer to reducing the threat to vulnerable wildlife in the region, and possibly also the spread of Ebola beyond the region, is to stop the illegal export trade in wildlife (dead or alive) on a regional or international scale. It is legitimate for other nations to intervene by preventing the import of African wildlife into their territories and supporting efforts to enforce local legislation banning the hunting and consumption of protected species. This seems a more equitable approach to addressing a culturally divisive issue: the consumption and trade in indigenous wild animals in particular regions of some developing countries. (It smacks a little of hypocrisy to ask African governments to forbid the use of local natural resources for human livelihoods.) At the same time, wildlife conservationists working in Africa still need to be sensitive to arguments that they care more about indigenous fauna and flora than they do about indigenous humans.

It is worth considering the difference between the perspectives of wildlife conservationists and animal rights advocates—Williams is Regional Director, Africa, for the World Society for the Protection of Animals. Many conservationists become attached to the species they work with, and to individual animals, but on the whole the discipline works at the level of the species to be conserved rather than the individual animal. Animal rights advocates, in contrast, believe that every individual animal deserves to be rescued from death or maltreatment. This universalizing perspective cuts across all considerations of political or cultural autonomy. In some ways, this is of course laudable, and we should recognize that animal rights organizations have contributed to raising the levels of care for animals (both wild and domesticated). However, to save every individual animal is impractical when it comes to managing wildlife and is in many places politically and culturally untenable.

Instead, there is much to be learned about the intertwined destinies of humans and wildlife in these regions. The study of Ebola, Marburg, and Lassa fevers and other zoonoses that cross over from animal reservoirs to humans needs to be shifted back further than the human–human contagion phase. We need to investigate the interactions between humans and infected wildlife. This could be effected more efficiently by more thorough investigations of landscape change and use by humans and animals and of their interactions from precolonial times to the present. For instance, seasonal migrations of fruit bats to the remnants of colonial‐era plantations and the accompanying supplementation of local livelihoods by the sale of hunted bats resulted in an Ebola outbreak in Luebo, Democratic Republic of Congo, in 2007 (Leroy et al. 2009).

Unravelling such outbreaks requires an understanding of the social and ecological dimensions of local landscapes, including seasonal aspects of human and (for example) bat behavior, livelihood concerns, and the culture and economics of hunting. Interdisciplinary research focused on mapping and investigating infection hotspots could provide a way forward, but it will require ecologists to engage with the human dimensions of the problem, social science and health workers to consider the ecological and biological dimensions of the crises (Brown & Kelly 2014), and environmental historians to provide a historical perspective on these social–ecological systems (e.g., McNeill 2010; Brown 2011).

Claiming that the 2014 Ebola epidemic has a “silver lining” for conservation in that it provides an opportunity to prevent the consumption of bushmeat (Williams 2014), and perhaps deforestation, is misguided. Framing this human tragedy in this way risks unforeseen conservation outcomes on the ground in Africa, and could alienate support for conservation efforts in the territories where it is most needed, as well as in the developed world (@vbadpanda 2014). Such large‐scale human disasters can at best be used as occasions to explain the important ways in which human and animal lifestyles are intimately interlinked and the consequences of these disasters for animal and human health.

## References

[cobi12454-bib-0001] @vbadpanda . 2014 How (some) conservationists are getting it wrong about Ebola. 20 October. Available from http://vbadpanda.wordpress.com/tag/bushmeat/ (accessed October 10, 2014).

[cobi12454-bib-0002] Bennett EL , et al. 2006 Hunting for consensus: reconciling bushmeat harvest, conservation, and development policy in West and Central Africa. Conservation Biology 21:884–887. 17531066 10.1111/j.1523-1739.2006.00595.x

[cobi12454-bib-0003] Brown H , Kelly AH . 2014 Material proximities and hotspots: toward an anthropology of viral hemorrhagic fevers. Medical Anthropology Quarterly 28:280–303. 24752909 10.1111/maq.12092 PMC4305216

[cobi12454-bib-0004] Brown K . 2011 Mad dogs and meerkats: a history of resurgent rabies in Southern Africa. Ohio University Press, Athens.

[cobi12454-bib-0006] Fa JE . 2007 Bushmeat markets: White elephants or red herrings? Pages 47–60 in Brown D, Davies G, editors. Bushmeat and livelihoods: wildlife management and poverty reduction conservation science and practice 2. Blackwell Publishing, Malden, MA.

[cobi12454-bib-0007] Fa JE , Brown D . 2009 Impacts of hunting on mammals in African tropical moist forests: a review and synthesis. Mammal Review 39:231–264.

[cobi12454-bib-0005] Fa JE , Currie D , Meeuwig J . 2003 Bushmeat and food security in the Congo Basin: linkages between wildlife and people's future. Environmental Conservation 30:71–78.

[cobi12454-bib-0008] Leroy E , Epelboin A , Mondonge V , Pourrut X , Gonzalez J‐P , Muyembe‐Tamfum J‐J , Formenty P . 2009 Human Ebola outbreak resulting from direct exposure to fruit bats in Luebo, Democratic Republic of Congo, 2007. Vector‐bourne and Zoonotic Diseases 9:723–728. 10.1089/vbz.2008.0167 19323614

[cobi12454-bib-0009] McNeill J . 2010 Mosquito empires: ecology and war in the greater Caribbean, 1620–1914. Cambridge University Press, Cambridge.

[cobi12454-bib-0010] Milner‐Gulland EJ , et al. 2003 Wild meat: the big picture. Trends in Ecology & Evolution 18:351–357.

[cobi12454-bib-0011] Nasi R , Taber A , Van Vliet N . 2011 Empty forests, empty stomachs? Bushmeat and livelihoods in the Congo and Amazon Basins. International Forestry Review 13:355–368.

[cobi12454-bib-0012] Osofsky S . 2014 How to keep viruses in the wild from finding humans. CNN. http://edition.cnn.com/2014/10/09/opinion/osofsky‐ebola‐wildlife/ (accessed October 9).

[cobi12454-bib-0016] Raymond M . 2014 Second pilot badger culls begin—NFU letter. National Farmers Union: The Voice of British Farming. Available from http://www.nfuonline.com/science‐environment/bovine‐tb/second‐pilot‐badger‐culls‐begin‐nfu‐letter/ (accessed September 9).

[cobi12454-bib-0013] Stern JE . 2014 Hell in the hot zone. Vanity Fair. Available from http://www.vanityfair.com/politics/2014/10/ebola‐virus‐epidemic‐containment (accessed October 10).

[cobi12454-bib-0014] WHO (World Health Organization) . 2014 WHO statement on the meeting of the International Health Regulations Emergency Committee regarding the 2014 Ebola outbreak in West Africa. Available from http://www.who.int/mediacentre/news/statements/2014/ebola‐20140808/en/ (accessed August 8).

[cobi12454-bib-0015] Williams T . 2014 Ebola's silver lining: we can clamp down on bushmeat. New Scientist 2985 (8 September).

[cobi12454-bib-0017] Young R . 2014 Take bushmeat off the menu before humans are served another Ebola. The Conversation 14 October. Available from http://theconversation.com/take‐bushmeat‐off‐the‐menu‐before‐humans‐are‐served‐another‐ebola‐32914.

